# Stress Driven Discovery of Natural Products From Actinobacteria with Anti-Oxidant and Cytotoxic Activities Including Docking and ADMET Properties

**DOI:** 10.3390/ijms222111432

**Published:** 2021-10-22

**Authors:** Syed Shams ul Hassan, Ishaq Muhammad, Syed Qamar Abbas, Mubashir Hassan, Muhammad Majid, Hui-Zi Jin, Simona Bungau

**Affiliations:** 1 Shanghai Key Laboratory for Molecular Engineering of Chiral Drugs, School of Pharmacy, Shanghai Jiao Tong University, Shanghai 200240, China; Shams1327@yahoo.com (S.S.u.H.); mishaqjnj@yahoo.com (I.M.); 2 Department of Natural Product Chemistry, School of Pharmacy, Shanghai Jiao Tong University, Shanghai 200240, China; 3 Department of Pharmacy, Sarhad University of Science and Technology, Peshawar 25000, Pakistan; qamar0613@yahoo.com; 4 Institute of Molecular Biology and Biotechnology, The University of Lahore, Lahore 54000, Pakistan; mubashirhassan_gcul@yahoo.com; 5 Department of Pharmacy, Capital University of Science and Technology, Islamabad 44000, Pakistan; 6 Department of Pharmacy, Faculty of Medicine and Pharmacy, University of Oradea, 410028 Oradea, Romania; simonabungau@gmail.com

**Keywords:** natural products, stress-technique, actinobacteria, ADMET, anti-oxidant, anti-cancer

## Abstract

Elicitation through abiotic stress, including chemical elicitors like heavy metals, is a new technique for drug discovery. In this research, the effect of heavy metals on actinobacteria *Streptomyces* sp. SH-1312 for secondary metabolite production, with strong pharmacological activity, along with pharmacokinetics profile, was firstly investigated. The optimum metal stress conditions consisted of actinobacteria strain *Streptomyces* sp. SH-1312 with addition of mix metals (Co^2+^ + Zn^2+^) ions at 0.5 mM in Gause’s medium. Under these conditions, the stress metabolite anhydromevalonolactone (MVL) was produced, which was absent in the normal culture of strain and other metals combinations. Furthermore, the stress metabolite was also evaluated for its anti-oxidant and cytotoxic activities. The compound exhibited remarkable anti-oxidant activities, recording the IC_50_ value of 19.65 ± 5.7 µg/mL in DPPH, IC_50_ of 15.49 ± 4.8 against NO free radicals, the IC_50_ value of 19.65 ± 5.22 µg/mL against scavenging ability, and IC_50_ value of 19.38 ± 7.11 µg/mL for iron chelation capacity and the cytotoxic activities against PC3 cell lines were recorded with IC_50_ values of 35.81 ± 4.2 µg/mL after 24 h, 23.29 ± 3.8 µg/mL at 48 h, and 16.25 ± 6.5 µg/mL after 72 h. Further mechanistic studies have revealed that the compound MVL has shown its pharmacological efficacy by upregulation of P53 and BAX while downregulation of BCL-2 expression, indicating that MVL is following apoptosis in varying degrees. To better understand the pharmacological properties of MVL, in this work, the absorption, distribution, metabolism, excretion, and toxicity (ADMET) were also evaluated. During ADMET predictions, MVL has displayed a safer profile in case of hepatotoxicity, cytochrome inhibition and also displayed as non-cardiotoxic. The compound MVL showed good binding energy in the molecular docking studies, and the results revealed that MVL bind in the active region of the target protein of P53 and BAX. This work triumphantly announced a prodigious effect of heavy metals on actinobacteria with fringe benefits as a key tool of MVL production with a strong pharmacological and pharmacokinetic profile.

## 1. Introduction

Microorganisms quickly adapt and retort to the variations in the availability and concentrations of metals within their harsh and dynamic habitat [1,2]. Microorganisms that live in a stressed environment attract much attention as potential new caves of therapeutically active compounds [3,4]. Contrary to insight, metals hamper secondary metabolite production; recent research has found that metals can stimulate or improve the action of potentially potent medically and nutraceutically relevant metabolites [1,5,6]. Thanks to the improvement of molecular biology and chemical biology techniques that imply that abiotic factors such as heavy metals may switch the production of natural compounds by modifying the attitude of secondary metabolism. The secondary metabolites produced by modern techniques like genome sequencing [7], abiotic or biotic stress [8], co-culture [9] and biosynthetic engineering proved to have enhanced pharmacological profiles compared to the compounds produced by the normal lab culture. Natural products have played an essential role in pharmacological and nutraceutical development [10,11,12] 

Latterly, the usage of drugs derived from natural origin has increased dramatically because of their high safety profile and potency compared to synthetic pharmaceuticals [13,14]. Even though microbes’ potential to originate novel scaffolds appears boundless, a few significant hurdles impede the biotransformation of molecules into drugs. One stumbling block in identifying secondary metabolites of these essential medicinal drugs is genes clusters or non-activated biosynthetic pathways. These gene clusters in this state are referred to as“sleeping gene clusters” [15,16]. The processes behind the metal-induced metabolite phenomena could be explained by the activation of sleeping genes or the production of molecules with a stereochemical affinity that enables metal complexation and transportation in biological systems [6,17].

Currently, a lot of researchers have been driven towards abiotic stress-discovery of natural products by using metals triggering plants [18,19], and microorganisms [20], as it is the fast, rapid and facile method for filtering out the novel compounds. Different researchers are focusing on multiple ways of research as some are focusing on increasing the secondary metabolites pattern [16], while some are untapping the microorganisms for their novel carbon scaffolds [6]. Our previous work produced an antibiotic by metal stress technique from microorganisms and enhanced its productivity by response surface methodology [5].

In this study, we used a metal-stress approach to explore the ability of the terrestrial actinobacteria *Streptomyces* sp. SH-1312 strain to elicit secondary metabolites in the presence of metal ions in the culture medium. Furthermore, the metal-elicited produced compound was evaluated for its anti-oxidant and cytotoxic activities, including Absorption, distribution, metabolism, excretion, and toxicity (ADMET).

## 2. Results

### 2.1. HPLC Evaluation of Secondary Metabolites of Metal Treated and Untreated Extracts

**Figure 1:**
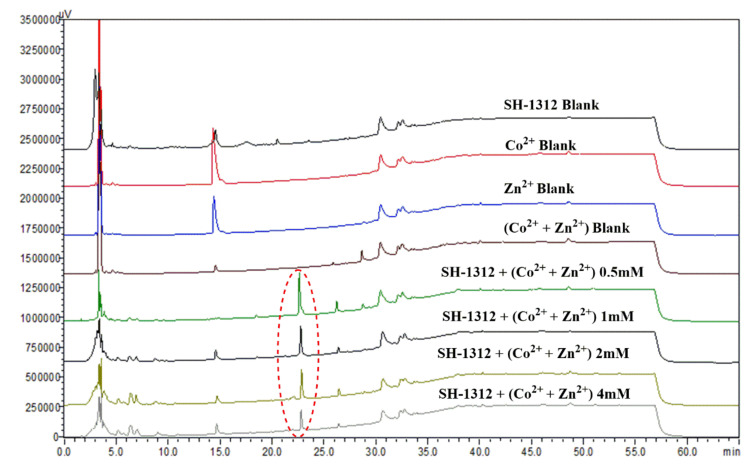
HPLC profile of metal treated and untreated SH-1312 strain with several mixed metals (Co^2+^ + Zn^2+^) ions. The red circle indicates the new peak in the HPLC chromatogram.

The impacts of elution mode, mobile phase, detection wavelength, and column temperature were conducted to determine the ideal HPLC setting for purification of the stressed metabolite of actinobacteria. Two different kinds of metals Co^2+^, Zn^2+^, and mix metals (Co^2+^ + Zn^2+^) were chosen as initial elicitors premised on their previous ability to produce secondary metabolites in microbes. The cultivation experiment of strain SH1312 was carried out in Gause’s medium having Co^2+^, Zn^2+^, and (Co^2+^ + Zn^2+^) ions with four different initial concentrations in a rotatory shaker at 180 rpm for 10 days at 28 °C. The two metals Zn^2+^ and Co^2+^ did not elicit any compound at any concertation, but a new peak was elicited in (Co^2+^ + Zn^2+^) (Figure 1). The alterations in the metabolic profile after subjecting metal ions were validated by establishing one medium without metal ions as a blank, three mediums without strain as a metal control, and four groups of media with distinct ionic concentrations (0.5 mM to 4 mM). A gauze filter separated mycelium and culture broth, and then the culture broth was extracted with EtOAc (2 × 200 mL). The HPLC chromatogram demonstrated that one specific stress-induced metabolite in the actinobacteria *Streptomyces* sp. (23.2 min for anhydromevalonolactone MVL) was detected in metal treated culture but was nearly undetectable in non-metal culture (Figure 1). The 0.5 mM concentration was shown to be the most effective among the various metal ion concentrations. Under normal cultivation conditions, the blank strain SH-1312 did not produce any metabolites (Figure 1). There was no peak at 23.2 min when actinobacteria strain SH-1312 was cultured alone in standard fermentation broth (Figure 1). A new peak appeared in the mix metals (Co^2+^ + Zn^2+^) stressed broth, triumphantly announcing that a stress metabolite was triggered by mix metals stress.

### 2.2. Identification and Structure Determination of Metal-Induced Secondary Metabolite

**Figure 2:**
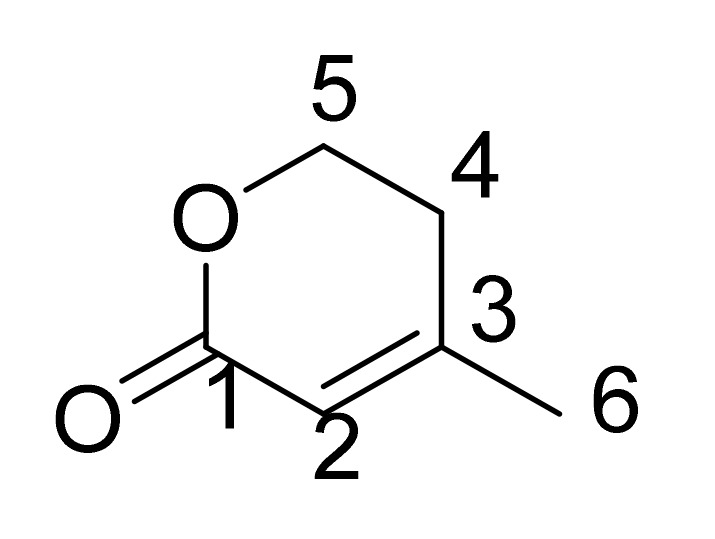
Chemical structure of anhydromevalonolactone.

Stress metabolite 1 was isolated as a white powder from the actinobacteria *Streptomyces* sp. SH-1312 with molecular formula C_6_ H_8_ O_2_. ^1^H NMR (CD_3_OD-*d_4_*, 500MHz) the proton NMR showed a total number of 4 signals δ2.04 (3H, s, H-6), δ2.46 (2H, t, *J* = 6.3, H-4), δ4.40 (2H, t, *J* = 6.3, H-5), δ5.80 (1H, d, *J* = 1.3, H-2) (Figure S1). ^13^C NMR (CD_3_OD-*d_4_*, 125MHz) the ^13^C NMR spectrum showed the presence of 6 signals, including 1 carboxyl group at δ166.1 (C-1), 1 quaternary carbon at δ160.6 (C-3) (Figure S2). DEPT135 shows a total no of 4 signals including one methyl (CH_3_) group at δ21.5 (C-6), one methane (CH) group at δ115.1 (C-2), and two methylene (CH_2_) groups at δ28.5 (C-4) and δ66.1 (C-5) (Figure S3). From the NMR data, stress metabolite was identified as a known compound anhydromevalonolactone (MVL) (Figure 2) [21]. This is the first example in which MVL was induced by metal elicitation.

### 2.3. Anti-Oxidant Assay

**Figure 3:**
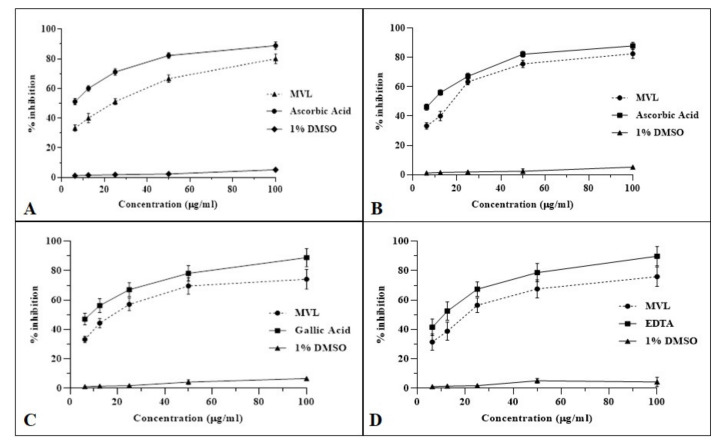
In vitro antioxidant activities of MVL. (**A**) DPPH radical scavenging activity, (**B**) NO scavenging activity, (**C**) OH● scavenging activity, and (**D**) iron-chelating % inhibition. Each value represents, Mean ± SD (*n* = 3).

To assess the antioxidant potential of MVL, multimode antioxidant assays were performed. In the DPPH assay, MVL showed promising results with 78.18 ± 4.2% inhibition of free radical at 100 µg/mL concentration compared to ascorbic acid (84.23 ± 2.9%) used as standard. IC_50_ for MVL in DPPH was recorded as 19.65 ± 5.7***µg/mL while for ascorbic acid 6.52 ± 4.92 µg/mL was recorded. MVL showed 80.05 ± 3.88% inhibition of NO free radicals in comparison to ascorbic acid (81.69 ± 2.69% inhibition) with IC_50_ of 15.49 ± 4.8**** for MVL and 8.44 ± 4.17 µg/mL for ascorbic acid. Similarly, MVL has shown significant scavenging ability against OH^●^ radicals and inhibited 72.69 ± 4.93% radicals at 100 µg/mL concentration compared to gallic acid (83.15 ± 3.67%) used as standard. IC_50_ for MVL and gallic acid was recorded as 19.65 ± 5.22*** and 6.26 ± 6.39 µg/mL, respectively. To gauge the chelation power of compounds to scavenge radicals, the iron chelation capacity of the compounds was evaluated. MVL was found to chelate 70.96 ± 5.79% (IC_50_ 19.38 ± 7.11***µg/mL) iron radicals in comparison to EDTA with 81.52 ± 4.67% chelation capacity and IC_50_ of 10.20 ± 6.54 µg/mL. All the observations are exhibited in (Figure 3) (Table 1).

### 2.4. Cytotoxicity Assessment

**Figure 4:**
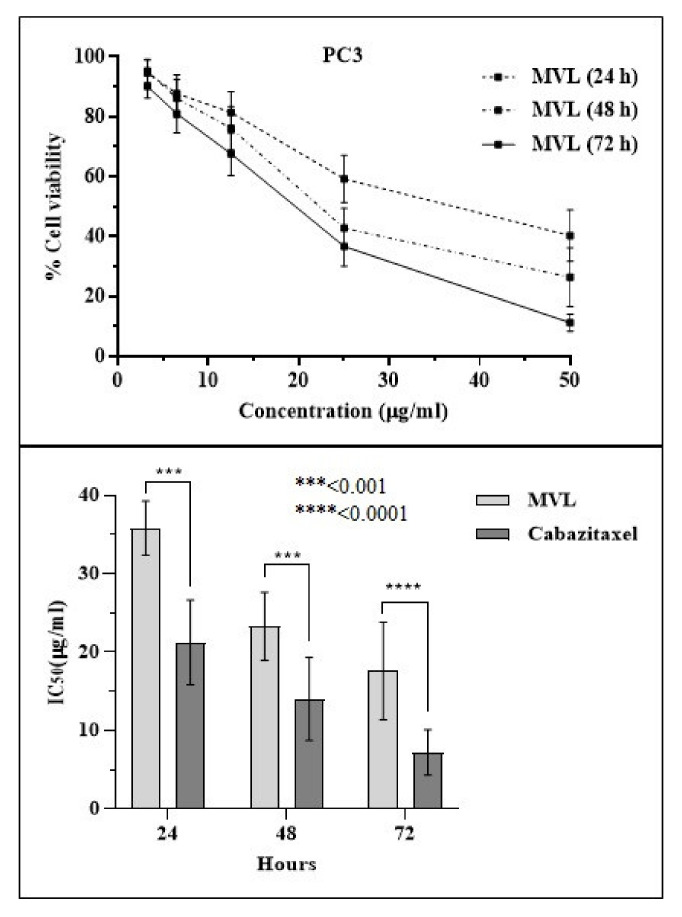
Effect of MVL on the viability of prostate cancer cells. MTT assay was used to determine the viability of cancer cells after 24, 48, and 72 h treatment of PC3 cells. Data is mean ± SEM of % cell viability (*n* = 3) at ***: *p* < 0.001 and ****: *p* < 0.0001.

To assess the cytotoxic potential of MVL against prostate cancer cell line (PC3), the MTT method was followed. Prostate cancer cells (PC3) were treated with multiple concentrations of MVL for 24 h, 48 h, and 72 h duration. Cabazitaxel was used as a standard drug. A significant decrease in cell viability has been examined on 24 h, 48 h, and 72 h exposure with MVL. MVL showed significant time-dependent inhibition in PC3 cell line with IC_50_ of 35.81 ± 4.2***µg/mL after 24 h, 23.29 ± 3.8****µg/mL at 48 h and 16.25 ± 6.5****µg/mL after 72 h treatment (Figure 4). While Cabazitaxel used as a standard drug exhibited an IC_50_ value of 21.16 ± 5.1 µg/mL after 24 h, 15.09 ± 5.7 µg/mL at 48 h and 9.25 ± 3.4 µg/mL after 72 h treatment (Figure 4, Table 2).

### 2.5. Cell Migration Assay

**Figure 5:**
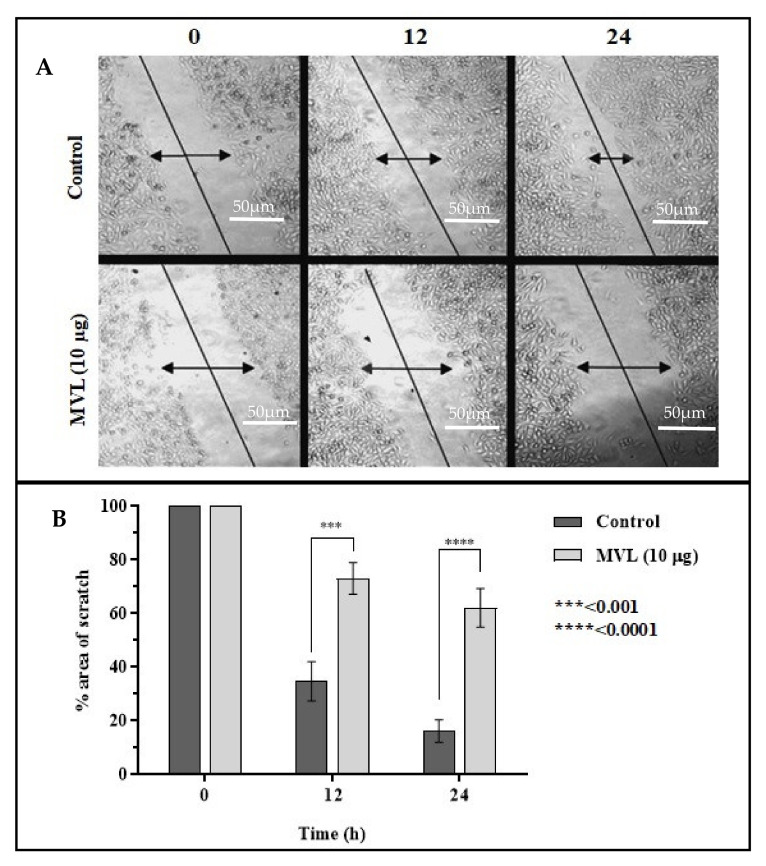
In vitro scratch assay on MVL treated prostate cancer cells. PC3 cells were plated in 6-well plates and scratched at the full confluence. Migration of cells to heal the area of the scratch was observed at 0 h, 12 h, and 24 h after treatment. Reduction in the area of the scratch was Photographed using Olympus CKX41 microscope and measured using ImageJ software. Data is mean ± SEM percent area of scratch in triplicate at ***: *p* < 0.001 and ****: *p* < 0.0001.

Stress compound MVL was tested for cell migration of PC3 using an established in vitro scratch test for 24 h. MVL (10 µg/mL) appeared to induce a significant reduction in cell migration at 12 and 24 h treatment of PC3. This was estimated by comparing the scratch area at every observation time as a percentage of control at 0 h. The area of scratch at 24 h treatment with MVL was recorded as 61.10 ± 5.2% compared to control (16.62 ± 4.4%) (Figure 5).

### 2.6. Molecular Expression Assessment

**Figure 6:**
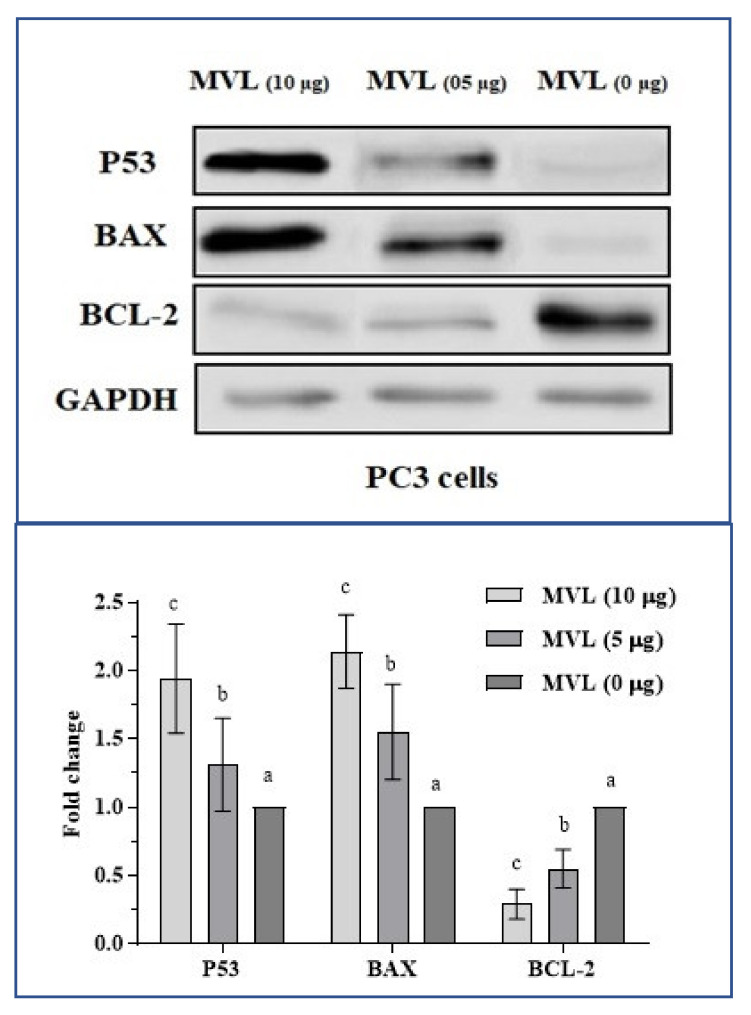
Western blot analysis of proteins associated with MVL induced apoptosis. Prostate cancer cells, PC3 were treated with MVL at a concentration of 0, 5, and 10 µg/mL for 48 h. Data shows increased expression of pro-apoptotic P53 and BAX while decreased expression of anti-apoptotic BCL2. GAPDH was used as a loading control. Fold change in P-53, BAX, and BCL-2 expression after treating for 48 h with different concentrations of MVL. Means with different superscripts (^a–c^) indicate significant (*p* < 0.001) difference in fold change between groups.

To unveil the possible mechanistic reason behind the anti-proliferation of cancer cell colonies in MTT assays, expression analysis of apoptotic proteins P53, BCL-2 and BAX were assessed via western blot technique (Figure 6). GAPDH served as a loading control. Prostate cancer cells (PC3) were treated with compound MVL at three concentrations (0, 5, and 10 µg/mL) for 48 h. Protein (40–60 µg) from protein lysate was segregated and probed with specified monoclonal antibodies. The upregulation of P53 and BAX while downregulation of BCL-2 expression indicates that MVL is following apoptosis in varying degrees. Fold change in protein expression after 48 h treatment is given in Figure 6.

### 2.7. Toxicity Assessment (Safety Profiling)

**Figure 7:**
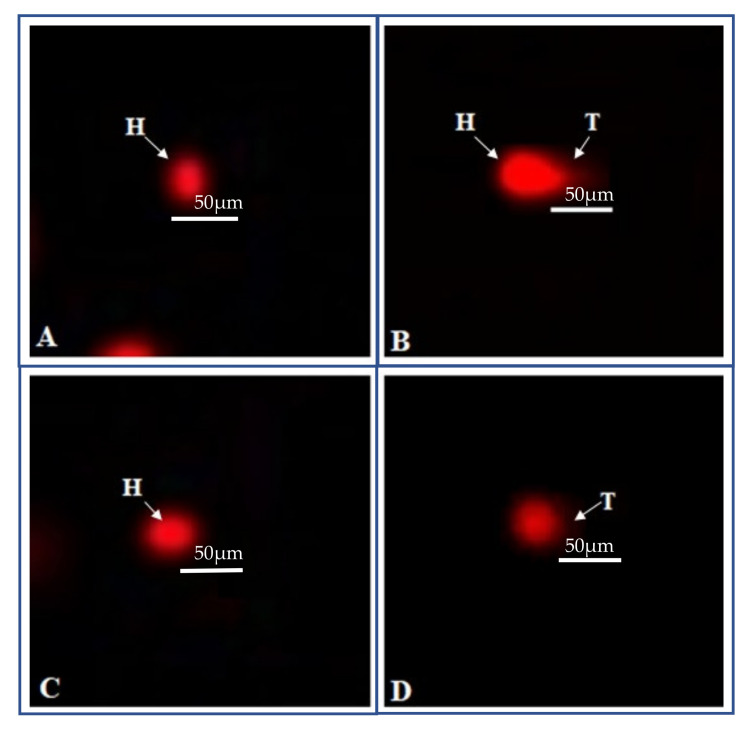
Genotoxicity evaluation of MVL on blood lymphocytes. “H” represents Head and “T” represents Tail. (**A**) Vehicle control (1% DMSO) (**B**) Ethyl methane sulfonate (20 µg/mL) (**C**) MVL (10 µg/mL) (**D**) MVL (20 µg/mL).

To evaluate toxicity, blood lymphocytes were treated with 10 and 20 µg/mL MVL samples, 20 µg/mL EMS (positive), 1% DMSO in PBS (negative control), and comet assay was performed. The slides were envisioned under a fluorescent microscope, and the extent of DNA damage was evaluated by analyzing the photomicrographs via CASP 1.2.3.b image analysis. Analysis of 50–100 cells from each sample was performed to assess comet length, head length, tail length, tail moment, DNA content in the head, and tail of lymphocytes. MVL (10 and 20 µg/mL) showed no significant damage to nuclear material compared to EMS used as standard genotoxic control. The tail length (18.5 ± 1.4 µm) of EMS-treated DNA clearly indicates the production of nicks in nuclear content, representing a greater extent of genotoxicity. While the stressed compound MVL showed no significant toxicity to DNA having the least tail content compared to control. All the fluorescent photomicrographs of the treated lymphocytes and their comet parameters are presented in Figure 7 and Table 3.

### 2.8. Pharmacokinetic and Toxicological Properties

Absorption, distribution, metabolism, excretion, and toxicity (ADMET) prediction studies were conducted for the stress compound MVL.

#### 2.8.1. Pharmacokinetic Properties

**Figure 8:**
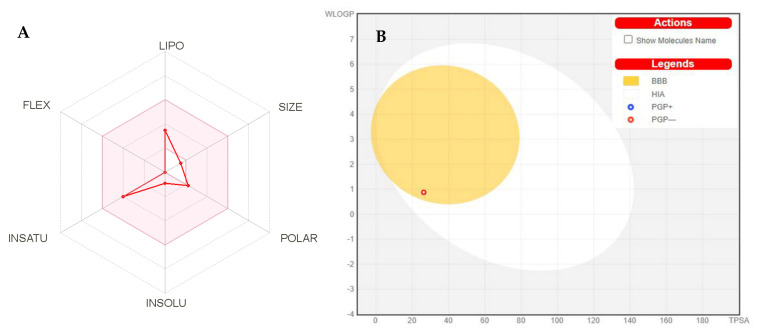
(**A**)**.** Bioavailability radar chart for MVL. The pink zone represents the physicochemical space for oral bioavailability, and the red line represents the oral bioavailability properties. (**B**) Predicted BOILED-Egg plot from swiss ADME online web tool for MVL.

The physicochemical properties of the compounds are mentioned in (Table 4). According to Table 4, the MVL physicochemical properties were analyzed and divided into 6 major groups with their suitable ranges for oral bioavailability (Figure 8a) encompassing lipophilicity (LIPO) 0.7 < (Log *P*_o/w_) XLOGP3 < +5.0, size (SIZE) 150 g/mol < MV < 500 g/mol, polarity (POLAR) 20Å^2^ < TPSA < 130 Å^2^, Insolubility (INSOLU) 0 < Log S (ESOL) < 6, Insaturation (INSATU) 0.25 < Fraction Csp3 < 1 and flexibility (FLEX) 0 < No. of rotatable bonds < 9. The Topological polar surface area (TPSA) scores of MVL were in the range of 20Å^2^ to 130Å^2^, suggesting MVL provides good transport properties in vivo. Figure 8a displays the oral bioavailability graph of the MVL based on the six factors discussed in physicochemical properties. The compound MVL has shown the results within these limits, displaying that MVL has a good physiochemical profile, one of the necessary parameters followed in pharmaceuticals or clinical trials.

HIA and CNS absorption are important parameters checked for every biomolecule before its entry for drug formulation in the pharmaceutical or clinical trials field [22]. The Blood-brain barrier penetration is essential as if the compounds that act on the central nervous system (CNS) must cross through the blood–brain barrier and the inactive compounds on the CNS should not intersect to avoid adverse effects on the CNS [23]. As mentioned in (Table 4) the stress compound MVL has displayed a high gastrointestinal absorption (HIA) with less BBB permeability.

Figure 8B displays the BOILED-EGG graph [24], which predicts the GI absorption (HIA) and BBB penetration of MVL. The white region is for GI (HIA) absorption zone and the yellow area (yolk) is for the BBB penetration zone. If any compound exists in the grey zone, it indicates that the compound is not absorbed nor BBB penetrant. The stress compound MVL displayed that it is not a P-gp (P-glycoprotein) substrate; therefore, MVL is not susceptible to the efflux mechanism of P-gp; many cancer cell lines utilize that as a drug resistance mechanism. Moreover, the compound MVL has shown less skin permeation Log *K*_p_ (Table 5), the more negative the *K*_p_, the less skin permeant the molecule is [25].

Furthermore, this tool also predicts five major cytochromes (CYP) isoforms. These enzymes play a crucial role in drug excretion, and these isoforms are metabolizing almost 75% of market available drugs. Inhibition of any of these isoforms results in causing some significant pharmacokinetics-based drug-drug interactions [25,26]. As mentioned in (Table 5) The stress compound MVL did not inhibit any cytochrome isoform and is quickly metabolized, which means that it cannot create any drug-drug interactions with these selected cytochromes. In the incidence of excretion, drug clearance is calculated as the sum of hepatic and renal clearances, and it is crucial for establishing dosing rates to reach steady-state concentrations. The clearance value of the MVL was inadequate. Organic Cation Transporter 2 (OCT2) substrates may impact adverse interactions with OCT2 inhibitors in combination. The compound MVL has been predicted as a non-substrate of OCT2.

#### 2.8.2. Toxicity Assessment

Before any drug enters the clinical trials phase or pharmaceutical industry manufacturing phase, it is imperative to consider the drug’s toxicology profile [22]. The stress compound MVL has been evaluated for its different kinds of toxicities, including human, oral rat, and environmental (Table 6). The Ames test is used to determine a compound’s mutagenic ability. According to the findings, MVL was categorized as non-Ames hazardous, indicating that they are unlikely to be carcinogens. Inhibition of the hERG-encoded potassium channels can result in catastrophic ventricular arrhythmia. According to the results, MVL suppresses both hERG I and hERG II. The compound MVL was also predicted as non-hepatotoxic, which indicates that it would not cause drug-induced liver injury.

In silico predictive model of median lethal dose (LD50) values for rats with oral administration was conducted by the General Unregulated Structure-Activity Relationships (GUSAR) programme (http://www.way2drug.com/gusar/acutoxpredict.htmL, accessed on 1 October 2021). Furthermore, for the prediction of lethal dose (LD50), the compound MVL had a score of more than 300 mg/kg and was classified as class 4; therefore, it is considered “harmful if swallowed” (300 < LD50 ≤ 2000) (Table 6). In addition to considering environmental toxicity, the online web server GUSAR was used. The online web server predicted the environmental toxicity, where 96-h fathead minnow 50% lethal concentration, 48-h *Daphnia magna* 50% lethal concentration, *Tetrahymena pyriformis* 50% growth inhibition concentration, and bioconcentration factors were evaluated. The results are depicted in Table 4. In the case of environmental toxicity prediction by GUSAR, MVL falls in the applicability domain of models in all cases. The toxicity profile shows that the stress compound MVL has a high safety profile, especially in hepatotoxicity.

#### 2.8.3. Cardiac Toxicity

**Figure 9:**
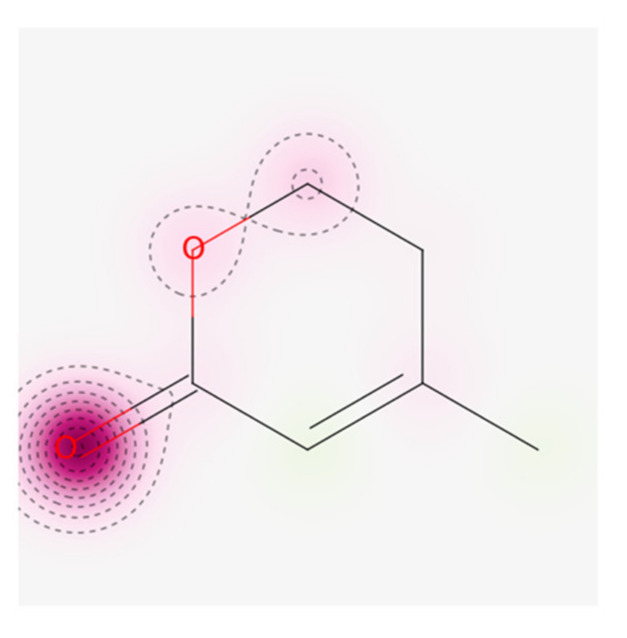
Map of Cardiac toxicity of MVL obtained from pred-hERG.

The blockage of the hERG K^+^ channels has been linked to fatal cardiac arrhythmias and before any biomolecule is elected as a drug candidate, it is mandatory by the FDA to check its hERG safety. The probability map of MVL derived after predicting cardiac toxicity by pred-hERG is given in Figure 9. Atoms or fragments’ positive and negative contributions to the hERG blockage have been found. The intense pink color means a negative contribution of an atom or fragment to the hERG blockage. According to the pred-hERG predictions, the stress compound MVL was predicted as non-cardiotoxic with a 70% confidence value. The results have demonstrated that MVL is safer for cardiac toxicity.

### 2.9. Molecular Docking

#### 2.9.1. Molecular Docking and Binding Energy Analysis

The docked complexes of MVL against BAX and P53 are formed and evaluated based on minimum energy values and ligand interactions patterns. Results showed that MVL showed good binding energy, value bind in the active region of the target protein (Table 7).

#### 2.9.2. Binding Analyses of MVL against BAX and P53

**Figure 10:**
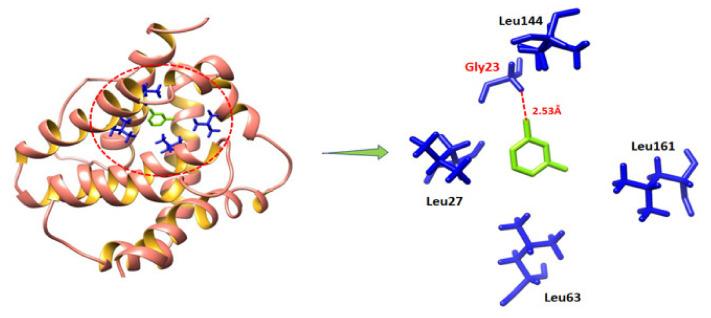
Binding analysis of MVL with BAX.

The generated docking complexes showed that MVL was confined in the active binding pocket of BAX, as mentioned in Figure 10. The results showed that a single hydrogen bond was observed in the screening process. The oxygen atom of the compound forms a hydrogen bond against Gly23 with a bond length of 2.53 Å. Prior data showed that BAX showed different binding pocket residues such as Tyr21, Ser24, His20, Asp29, Leu23, and Ile19 are more important for activating downstream signaling pathways. Our docking results showed that ligand binds within the target protein’s active site, which may involve the cascade of regulatory signaling proteins [27].

**Figure 11:**
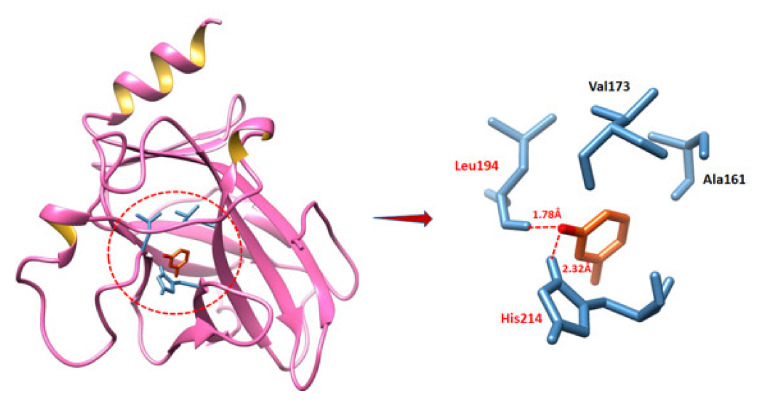
Binding analysis of P53 with MVL.

In P53 docking, MVL is confined in the active binding pocket of the target protein, as mentioned in Figure 11. The generated docking results showed that a couple of hydrogen bonds were observed at different residues positions. An oxygen atom of the compound formed hydrogen bonds with Leu194 and His214 with bond distances 1.78 Å and 2.32 Å, respectively. Leu194 and His214 are binding pocket residues that may involve in the downstream signaling pathways. Our docking results showed that MVL is directly bound with active site residues. Prior docking studies also showed a good correlation with our predicted docking results [28].

## 3. Discussion

This research focused on isolating a natural product MVL as a stressed compound from actinobacteria by abiotic stress technique. Furthermore, evaluating the in vitro pharmacological profile of MVL as an anti-oxidant and anti-cancer agent, including In-silico studies of ADMET and molecular docking, strengthened its profile.

The metal-stress technique opens the road towards the most promising directions and increases the chances for novel secondary metabolite production. As proved from the previous findings, stress-elicited compounds are generally absent in normal culture method but after inducing abiotic stress the new compound with its unique carbon skeletons appeared in stressed culture [1,5,17]. The microorganisms sometimes show resistance towards the metals and therefore need to either increase the concentration of metals or to use the combination of two or more metals to trigger the sleeping genes [1]. Our research shows that the actinobacteria strain initially didn’t respond to any metal in its single entity. However, after combining two metals the actinobacteria responded to the metals ions in its environment and elicited a new peak as MVL.

Nowadays, many natural products researchers have driven their research towards untapping the secondary metabolites with promising anti-oxidant and anti-cancer properties [11,13,29,30]. As mentioned in Table 1 the MVL has shown promising antioxidant properties with recording the IC_50_ value of 19.65 ± 5.7 µg/mL in DPPH, IC_50_ of 15.49 ± 4.8 against NO free radicals, the IC_50_ value of 19.65 ± 5.22 against scavenging ability, and IC_50_ value of 19.38 ± 7.11µg/mL for iron chelation capacity. These results clearly indicate that MVL has a robust anti-oxidant profile. Furthermore for the cytotoxic activities as mentioned in Table 2. The compound MVL has shown promising results against PC3 cell lines with IC_50_ values of 35.81 ± 4.2 µg/mL after 24 h, 23.29 ± 3.8 µg/mL at 48 h, and 16.25 ± 6.5 µg/mL after 72 h. To achieve the mechanistic studies for the compounds’ pharmacologic profile it was observed that MVL has shown its cytotoxic response by upregulation of P53 and BAX while downregulation of BCL-2 expression, indicating that MVL is following apoptosis in varying degrees.

ADMET is a crucial stage for every kind of biomolecule before its biotransformation into a drug [22]. According to the ADMET profile of MVL, its absorption and distribution were moderate; MVL is highly soluble in GIT with less BBB permeability which can show that MVL cannot create any serious adverse effects related to CNS. As the inactive compounds on the CNS should not intersect to avoid adverse effects on the CNS [23]. Furthermore, MVL has revealed that it is not a P-gp (P-glycoprotein) substrate; therefore, MVL is not susceptible to the efflux mechanism of P-gp, many cancer cell lines utilize that as a drug resistance mechanism. CYP enzymes play a crucial role in drug excretion, and these isoforms are metabolizing almost 75% of market available drugs. Inhibition of any of these isoforms results in causing some significant pharmacokinetics-based drug-drug interactions [25,26]. MVL has not inhibited any CYP enzymes, which means MVL cannot create drug-drug interactions for those CYP enzyme-targeted drugs. One of the significant drawbacks of many drugs is to create hepatotoxicity [31] and cardiotoxicity [32]. MVL has not shown any hepatotoxicity proved to be non-cardiotoxic with a 70% confidence value. The hERG K+ channel blockade can contribute to QT prolongation and possibly life-threatening arrhythmia [33]. Therefore, MVL was expected to be a non-inhibitor of hERG and will not cause any cardiac side effects. Finally, as derived from pkCSM, the toxicity profile was optimal. Acute toxicity is described as the adverse effects of a single reaction to a drug during a predefined timeframe [34]. In general, mice and rats are used to measure acute toxicity. MVL was expected to be non-toxic and classified in class four with harmful indications if swallowed, suggesting a safer application. Environmental toxicity assessment is more applicable to pesticides or similar compounds. MVL did not exhibit any environmental toxicity violations.

As evident from our study, MVL has shown encouraging results in terms of p53 and BAX inhibition in the sense of molecular docking. Therefore, in these ways, special attention should be placed on investigating this process’s therapeutic importance.

Thus, the current study revealed the potential advantages of Stress driven compound MVL and may be useful against different diseases. The research relied on in vitro and computational tools that documented pharmacological properties and bioactivities predictions. Moreover, clinical studies are necessary to confirm the findings of the present work. Nonetheless, the results of this work will provide future guidance for the design and development of new lead compounds as anti-oxidant and anti-cancer agents.

## 4. Materials and Methods

### 4.1. General

High-performance liquid chromatography (HPLC) system was used with a set of Waters 996 Photodiode Array Detector and a Waters 717 plus Autosampler (Waters, Shinagawaku, Tokyo, Japan). On a Bruker AVANCE DMX 500 NMR spectrometer with TMS as an internal standard, 1H NMR (500 MHz) and 13C NMR (125 MHz) spectra were measured at 25 °C.

### 4.2. Soil Sample Collection

Soil sediment samples were obtained from the lower Orakzai Agency, Pakistan, at 300 feet in the bushy mountains. The sediments were collected in unpolluted areas near the lower Orakzai Agency in Pakistan. The samples were then taken to the lab and kept at 4 °C in the refrigerator.

### 4.3. Isolation and Storage of SH-1312 Strain

To obtain a 10^−1^ dilution, the fresh soil sample of about 1–2 g was immediately subjected to the pre-sterilized glass vials and diluted with simulated seawater. The mixture was sonicated for 1 min to liberate microorganisms tied to the soil particles, then shaken for 15 min at room temperature. The serial dilutions of up to10^−2^, 10^−3^, and 10^−4^ were then prepared. To prevent fungal contamination, nystatin (0.05 g/L) was added to the pre-prepared isolation media (Gause’s synthetic agar). Following the preparation of the dilutions, 100 µL aliquots of each dilution were inoculated on each media and disseminated with the pre-sterilized spreader. For 15–20 days, the plates were incubated at 28 °C.

Purified colonies of actinobacteria were collected and kept on agar media and stored at 4 °C. The actinobacteria were identified by their morphology, most commonly their colors. Based on the ITS 16S segment, the SH-1312 strain was recognized as a *Streptomyces* sp.

### 4.4. Metal Stress and Normal Cultivation

The metal stress and normal cultures of strain SH-1312 were conducted simultaneously in 500 mL flasks containing 200 mL liquid Gause’s medium for ten days at 28 °C in a rotatory shaker at 180 rpm. As a control, a standard broth culture of strain SH-1312 was conducted in two flasks. Additional 0.5 mM, 1 mM, 2 mM, and 4 mM/L CoCl_2_, ZnSO_4_, and (CoCl_2_ + ZnSO_4_) were added to the stressed culture media for SH-1312. The mycelium was withdrawn, and the culture broth was extracted twice with EtOAc in an equal volume.

### 4.5. HPLC Analysis and Purification of Stress Metabolite

The analysis of isolated compounds was done through a reversed-phase HPLC-UV equipped with a C18 column. The isolation procedure was set on an HPLC with an H_2_O/MeOH gradient from 20–100% for 0–30 min, 100% MeOH from 30–50 min with a constant flow rate of 0.8 mL/min using 210 wavelengths. The stress-induced metabolite was purified on preparative HPLC by setting a constant mobile phase of 70% MeOH for 28 min with a 9 mL/min flow rate.

### 4.6. Extraction and Isolation

EtOAc (2 × 200 mL) was used to extract the 13 L of fermented broth. After the solvent had evaporated, the crude residue was dissolved in methanol and centrifuged at 120,000 rpm for ten minutes before being exposed to analytical HPLC. After subjecting to the HPLC the initial screening was conducted by setting an H_2_O/MeOH gradient from 20%–100% for 0–30 min. As mentioned in Figure 1 the new peak was observed at 23.2 min. After that, the compound was purified on Preparative HPLC by a constant mobile phase of 70% MeOH, “MeOH: H_2_O (70:30)”. The purified compound was then subjected to its 1D NMR (^1^H NMR, ^13^C NMR, and DEPT135). The structure of the stressed compound Anhydromevalonolactone (MVL) (7.9 mg, tR = 17 min) was identified by 1D NMR.

### 4.7. Anti-Oxidant Evaluation

To evaluate the antioxidant potential of the stress compound, a multifaceted antioxidant evaluation of a stress compound was performed via DPPH scavenging assay, OH● radical scavenging assay, NO scavenging assay, and Iron chelating assay by following pre-described protocols [35].

### 4.8. Cytotoxicity Analysis against Human Prostate PC3 Cell Line

MTT assay was used to assess cytotoxicity against the human prostate PC3 cell line [30]. Non-adherent PC3 cells were cultured in a humidified CO2 incubator at 37 °C in complete growth medium RPMI 1640 enriched with 2.2 g/L NaHCO3, 100 g/mL streptomycin sulfate, 10% *v*/*v* heat-inactivated fetal bovine serum (HIFBS), 0.25 g/mL amphotericin B, and 100 IU/mL penicillin G sodium. 20 µL of a compound in 1% DMSO in PBS and 180 µL of PC3 cancer cells were combined in a 96-well plate to reach a final concentration of 20 µg/mL for evaluating the cytotoxicity on PC3 cancer cells. The cells were loaded at an assay density of 1 × 104 cells/mL. The plate was incubated in humidified 5% CO2 at 37 °C for 24 h, 48 h, and 72 h. Cabazitaxel was utilized as a positive control, and 1 percent DMSO in PBS was employed as a negative control. Cabazitaxel was procured from Enzo life sciences, Fisher Scientific (Thermo Fisher Scientific, California, USA) CAS-183133-96-2 (ENZCHM2170010). A stock solution (4 mg/mL) of Cabazitaxel was prepared in DMSO and diluted in 1× PBS to make it 1% DMSO. Then serial dilutions of the standard compound were prepared in such a way that wells received 50, 25, 12.5, 6.25, and 3.12 µg/mL of Cabazitaxel with ≤1% DMSO. After that, 20 µL of pre-filter sterilized MTT solution was added to plates and incubated at 37 °C for 4 h in a CO2 incubator. Colored formazan crystals were detached by deliberately removing the supernatant and dissolved in 100 µL of DMSO with absorbance measured at 540 nm using Elx 800 microplate reader. Cytotoxicity was calculated using the following formula:

% *cytotoxicity* = [100−(*As*/*Ac* × 100)] (1)

where *As* and *Ac* show the absorbance of sample and negative control, respectively. Cytotoxicity was calculated with GraphPad Prism 5 software.

### 4.9. In Vitro Wound Assay

In vitro wounds were induced by a modified protocol described [36], and the scratch assay was performed on cells to study the effect of stress compound on cell migration [15]. In the growth medium of 24-well plates, Human fibroblast cells were injected at a density of 5 × 104 cells/well. After achieving about 100% proliferation, the cells were shifted to the basal media for 24 h. The cells were scraped with a sterile micro pipetting tip after they had grown into a monolayer. A glass slide was slotted across the top of the dish to help keep the tip stationary when scratching and allow for a straight scratch. The tip was firmly dragged across the dish’s diameter. PBS was used to wash the cells to remove the loose debris. For each dose, a set of four wells were filled with different concentrations (1–40 g/mL) of a stressed compound. As a control, the cells were granted an equal amount of DMSO. For the migration assay, cultures were rinsed twice with PBS, preserved with absolute methanol, stained with Giemsa, and inspected at a magnification of 40× using a light microscope with a calibrated ocular. Images were recorded instantly after being wounded and then again portrayed at 0, 12, and 24 h time points. Using Image J software, cell migration rates were quantified by measuring the wound area change (pixels).

### 4.10. Western Blotting

PC3 cells were cultured in a T75 flask (1 × 106/flask). After 48 h, cells were treated with NA (20 µM and 40 µM) for 12 and 24 h. Following treatment, media was aspirated and cells were washed with cold PBS whose pH was maintained at 7.4 trypsinized and pelleted in 15 mL falcon tubes, and cold lysis buffer was added to the pellet. Protein extraction and western blot analysis were done by following the protocol described previously [30].

### 4.11. Comet Assay

DNA mutilation was evaluated by following the protocol as described by Peter et al. [37]. To remove dust and machine oil, the slides were soaked in methanol and then burned over a blue flame. Approximately three-quarters of the sterilized slides were dipped in a 1% solution of normal melting point agarose (NMPA) and allowed to place at room temperature. In 1 mL cold lysing solution, a puny segment of liver tissue was sliced into minuscule pieces. The diced tissue was mixed with 85 µL of low melting temperature agarose solution before coating on the pre-decorated slides. The slide was delicately covered with a coverslip and placed on ice packs for 10–12 min. By removing the coverslip, the second layer of low melting agarose was poured on the slide. After that, it was allowed to solidify on ice packs. After the third time coating the slide with low melting agarose, it was immersed in a lysing solution for 10 min before being placed in the freezer for roughly 2 h. After electrophoresis, the samples were stained with ethidium bromide (1%) and observed under a fluorescence microscope. For image analysis, CASP 1.2.3.b software was used to assess the extent of DNA damage. Nearly 50–100 cells were analyzed in each sample for head length, comet length, tail moment, tail length, and the amount of DNA in the nuclei of liver cells.

### 4.12. ADMET Analysis

ADMET (Absorption, distribution, metabolism, excretion, and toxicity) are the essential measurement tools for any compound before being elected as a drug candidate. The online web tool swiss ADME (http://www.swissadme.ch/index.php, accessed on 1 October 2021) was used to obtain ADMET properties of the stress compound [25], and Online web tool pkCSM (http://biosig.unimelb.edu.au/pkcsm/prediction, accessed on 1 October 2021) was used to predict the pharmacokinetic scores.

### 4.13. Prediction of Cardiac Toxicity

Blockage of the hERG K^+^ channels has been linked to fatal cardiac arrhythmias. To predict cardiac toxicity, a free accessible online service pred-hERG 4.2 (http://predherg.labmol.com.br, accessed on 1 October 2021) was used for its early detection of potential hERG blockers and non-blockers [38].

### 4.14. Molecular Docking

Repossession of BAX and P53 from PDB 

The crystal structure of BAX and P53 was retrieved from the Protein Data Bank (PDB) having PDBIDs 2LR1 and 4MZI, (www.rcsb.org, accessed on 1 October 2021), respectively. Energy minimization of target structures were carried out by using conjugate gradient algorithm and amber force field in UCSF Chimera 1.10.1 [22].

#### Designing of Ligand and Molecular Docking Simulation Using Autodock

The ligand was sketched in drawing ACD/ChemSketch, converted into PDB using the PyMOL tool, and minimized by visualizing software UCSF Chimera 1.10.1. PyRx docking tool was used to perform a molecular docking experiment for the ligands against the protein (BAX and P53). The grid box center values of (center_ X = 2.5557, −18.1355, center_ Y= 0.1669, −5.8255, and center_ Z = 2.35430, 20.40572) and size values were adjusted as (X = 20.2138, 26.6372, Y = 24.8519, 23.72620, and Z = 20.7404, 21.5794) for BAX and P53, respectively for better conformational position in the active region of target proteins. The ligand was docked separately against BAX and P53 with a default exhaustiveness value = 8. The predicted docked complexes were evaluated based on the lowest binding energy (Kcal/mol) values and structure-activity relationship (SAR) analyses. The three-dimensional (3D) graphical depictions of all the docked complexes were accomplished by Discovery Studio (2.1.0) and UCSF Chimera 1.10.1 [22].

## 5. Conclusions

The secondary metabolite patterns of an actinobacterial strain can alter under the presence of heavy metals supplemented to the fermentation medium, as proven by two chemical and pharmacological screening approaches. This work created a simple, quick, and easy approach for screening such kinds of secondary metabolites that were totally absent at the normal culture under optimal conditions. Strains isolated by the metal stress technique with enhanced pharmacological spectrum open a new road for the researchers to re-screen existing microbial strain libraries for unique secondary metabolites that emerged under the effects of heavy metals. The findings of this study triumphantly revealed that stress-driven discovery of potent biomolecule from the actinobacteria is an efficient technique for uncovering the microorganisms’ undiscovered pool of small molecules.

## Figures and Tables

**Table 1:** Assessment of in vitro antioxidant potential (IC_50_: µg/mL) of MVL.

Sample		IC_50_ (µg/mL)		
	DPPH Scavenging	NO Inhibition	OH● Inhibition	Iron Chelation
MVL	19.65 ± 5.7 ***	15.49 ± 4.8 ****	19.65 ± 5.22 ***	19.38 ± 7.11 ***
Ascorbic acid	6.52 ± 4.92	8.44 ± 4.17	--	--
Gallic acid	--	--	6.26 ± 6.39	--
EDTA	--	--	--	10.20 ± 6.54
1% DMSO	--	--	--	--

Note: Values are presented as mean ± SD of triplicate analysis, where ***: *p* < 0.001 and ****: *p* < 0.0001.

**Table 2:** Cytotoxicity assessment of MVL against prostate cancer cells (PC3).

Compound	IC_50_ (µg/mL)		
	24 h	48 h	72 h
MVL	35.81 ± 4.2 ***	23.29 ± 3.8 ****	16.25 ± 6.5 ****
Cabazitaxel	21.16 ± 5.1	15.09 ± 5.7	9.25 ± 3.4

Note: Values are presented as mean ± SD deviation of triplicate analysis, where ***: *p* < 0.001 and ****: *p* < 0.0001.

**Table 3:** Genotoxicity assessment of MVL on blood lymphocytes by comet parameters.

Sample	Comet Length (µm)	Head Length (µm)	Tail Length (µm)	% DNA in Head	% DNA in Tail	Tail Moment (µm)
Control	40.4 ± 4.2	35.9 ± 1.8	4.5 ± 0.5	88.8 ± 2.1	11.2 ± 1.3 ^β^	0.11 ± 0.04 ^β^
EMS (20 µg/mL)	42.6 ± 3.6	24.1 ± 2.7	18.5 ± 1.4	56.6 ± 3.5	43.4 ± 1.8 ^¥^	1.37 ± 0.11 ^¥^
MVL (10 µg/mL)	42.6 ± 3.1	36.7 ± 2.5	5.9 ± 0.3	86.1 ± 2.8	13.9 ± 1.8 ^β^	0.11 ± 0.03 ^β^
MVL (20 µg/mL)	41.4 ± 2.4	31.7 ± 1.8	8.5 ± 1.3	80.6 ± 1.8	19.4 ± 2.6 ^β, ¥^	0.24 ± 0.03 ^β, ¥^

Values are expressed as Mean ± SD (*n* = 3). Means with symbol “β” indicates non significant difference from control. “¥” from EMS group according to Kruskal-Wallis test at *p* < 0.05.

**Table 4:** Predicted physicochemical parameters and lipophilicity properties of MVL.

Properties	Parameters	MVL
Physicochemical properties	MW ^a^ (g/mol)	112.13
	Rotatable bonds	0
	HBA ^b^	2
	HBD ^c^	0
	Fraction Csp3	0.50
	TPSA ^d^	26.30
Lipophilicity Log *P_o/w_*	iLOGP	1.54
	XLOGP3	0.60
	MLOGP	0.88
	Consensus	1.07

^a^ Molecular weight, ^b^ H-bond acceptor, ^c^ H-bond donor, ^d^ Topological polar surface area.

**Table 5:** Predicted ADME parameters of MVL.

Properties	Parameters	MVL
Absorption	Water Solubility	−0.509
	GI ^a^	100
	Log *K*_p_ (Skin permeation) cm/s	−6.56
	P-gp substrate	No
Distribution	BBB ^b^	−0.031
	CNS permeation (Log PS)	−2.633
	V_D_^c^ (human)	−0.037
Metabolism	CYP1A2 inhibitor	No
	CYP2C19 inhibitor	No
	CYP2C9 inhibitor	No
	CYP2D6 inhibitor	No
	CYP3A4 inhibitor	No
Excretion	Total Clearance (log mL/min/kg)	0.814
	Renal OCT2 substrate	No

^a^ Gastrointestinal, ^b^ Blood–brain barrier, ^c^ Volume of distribution.

**Table 6:** Predicted Human, Oral rat, and Environmental toxicity profile of MVL.

Toxicity	Parameters	MVL
Human	Ames toxicity	No
	hERG I inhibitor	No
	hERG II inhibitor	No
	Hepatotoxicity	No
	Max. tolerated dose (human) (log mg/kg/day)	1.01
Oral Rat	Oral Toxicity (LD50) (mg/kg)	1890
	Oral Toxicity classification *	IV
*Environmental*	*Daphnia magna* LC50 -Log10 (mol/L)	3.347
	Bioaccumulation factor Log10 (BCF)	0.487
	*Tetrahymena pyriformis* IGC50 -Log10 (mol/L)	−0.867
	Fathead Minnow LC50 Log10 (mmol/L)	0.452

* Class I: fatal if swallowed (LD50 ≤ 5); Class II: fatal if swallowed (5 < LD50 ≤ 50); Class III: toxic if swallowed (50 < LD50 ≤ 300); Class IV: harmful if swallowed (300 < LD50 ≤ 2000); Class V: may be harmful if swallowed (2000 < LD50 ≤ 5000) and Class VI: non-toxic (LD50 > 5000).

**Table 7:** The binding energy of stressed compound MVL.

Docking Complexes	Binding Energy (Kcal/mol)
P53_Ligand	−5.6
BAX_Ligand	−6.7

## Supplementary Materials

The following are available online at https://www.mdpi.com/article/10.3390/ijms222111432/s1.
