# Supplementary material for: Stress Driven Discovery of Natural Products From Actinobacteria with Anti-Oxidant and Cytotoxic Activities Including Docking and ADMET Properties
Source: Int J Mol Sci. 2021 Oct 22;22(21):11432. doi: 10.3390/ijms222111432 (PMC8584265; doi:10.3390/ijms222111432)
Supplement: Supplementary file 1 [file ijms-22-11432-s001.zip › ijms-1408140-supplementary.pdf]

Stress Driven Discovery of Natural Products From Actinobacteria With Anti-Oxidant and Cytotoxic Activities Including Docking and ADMET properties

Syed Shams ul Hassan [1,2], Ishaq Muhammad [1,2], Syed Qamar Abbas [3], Mubashir Hassan[4], Muhammad Majid[5]*, Hui-Zi Jin[1,2]*, Simona Bungau[6]

1 Shanghai Key Laboratory for Molecular Engineering of Chiral Drugs, School of Pharmacy, Shanghai Jiao Tong University, Shanghai, 200240, PR China; Shams1327@yahoo.com ,

2 Department of Natural Product Chemistry, School of Pharmacy, Shanghai Jiao Tong University, Shanghai,200240, PR China

3 Department of Pharmacy, Sarhad University of Science and Technology, Peshawar, Pakistan; qamar0613@yahoo.com

4 Institute of Molecular Biology and Biotechnology, The University of Lahore, Pakistan; mubashirhassan_gcul@yahoo.com

5 Department of Pharmacy, Capital University of Science and Technology, Islamabad, Pakistan; (majidpharma808@gmail.com)

6 Department of Pharmacy, Faculty of Medicine and Pharmacy, University of Oradea, 410028 Oradea, Romania; (Simonabungau@gmail.com)

* Correspondence: Kimhz@sjtu.edu.cn (H.Z.J), Majidpharma808@gamil.com (M.M),

Supplementary data

Fig.S1. ^{1}HNMR spectrum of MVL
Fig.S2. ^{13}C NMR spectrum of MVL
Fig.S3. DEPT 135 spectrum of MVL

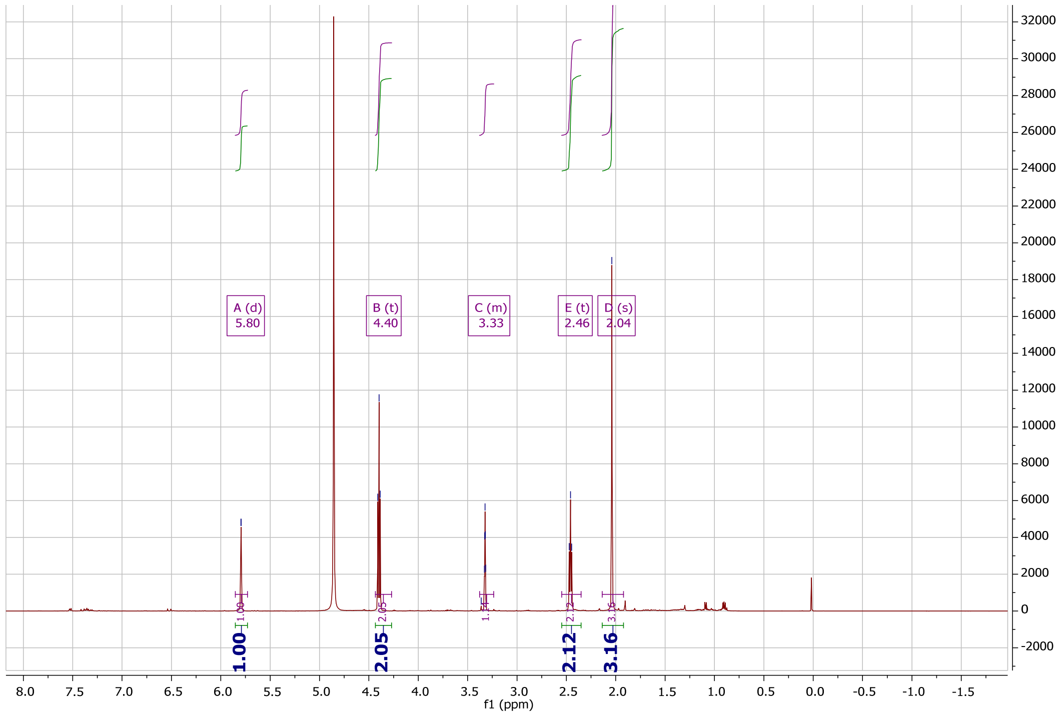

Fig.S1. ^{1}HNMR spectrum of MVL

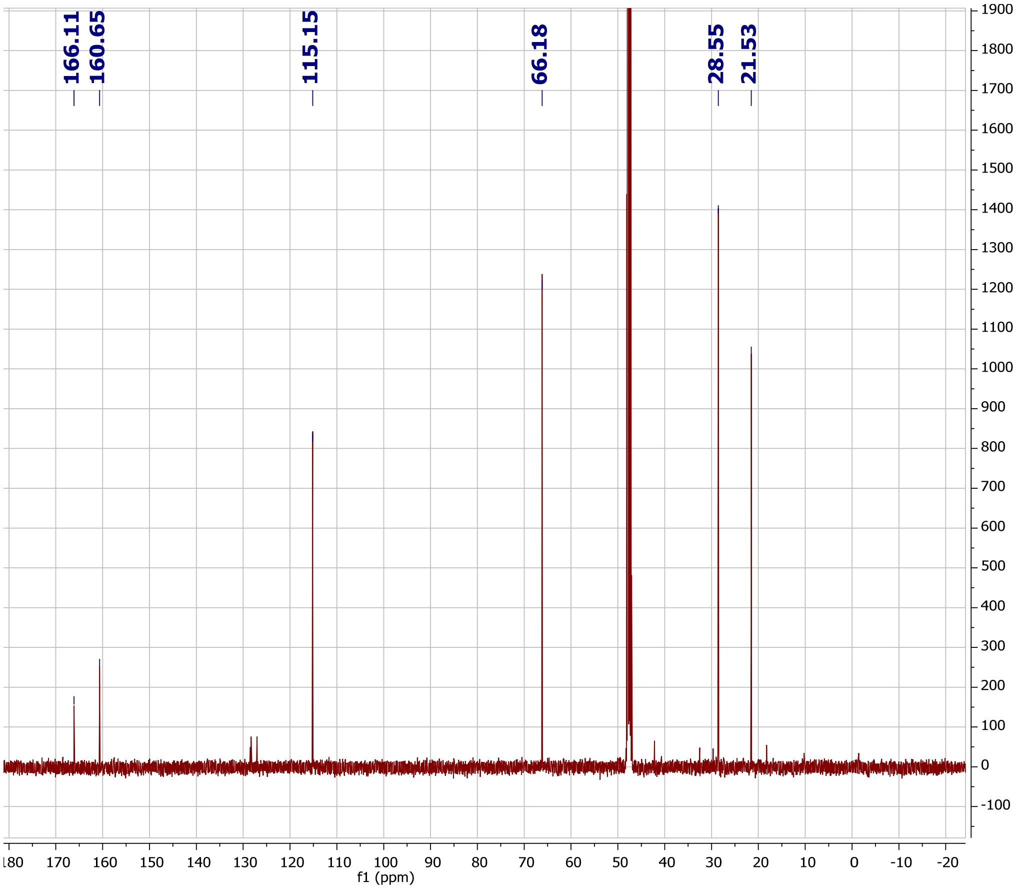

Fig.S2. ^{13}C NMR spectrum of MVL

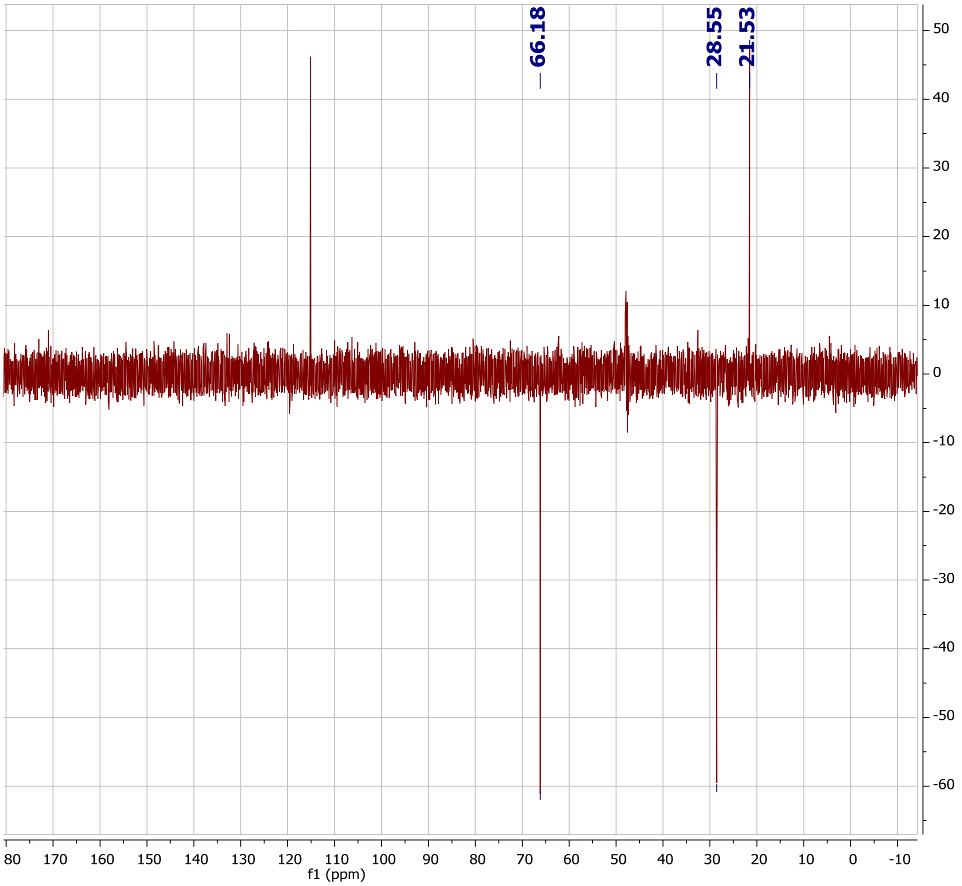

Fig.S3. DEPT 135 spectrum of MVL
